# Psychometric properties of the single-item measure, severity of worst tiredness, in patients with moderately to severely active rheumatoid arthritis

**DOI:** 10.1186/s12955-017-0807-5

**Published:** 2017-12-06

**Authors:** Elizabeth D. Bacci, Amy M. DeLozier, Chen-Yen Lin, Carol L. Gaich, Xiang Zhang, Terence Rooney, Stephanie de Bono, Richard Hoffman, Kathleen W. Wyrwich

**Affiliations:** 1 Evidera, Inc., 1417 4th Avenue, Suite 510, Seattle, WA 98101 USA; 2 Eli Lilly and Company, Indianapolis, IN USA; 3 Evidera Inc., Bethesda, MD USA

**Keywords:** Rheumatoid arthritis, Tiredness, Fatigue, Patient-reported outcome, Psychometric, Reliability, Validity, Responsiveness

## Abstract

**Background:**

To assess the reliability, validity, and responsiveness to treatment change of the single-item measure, Severity of Worst Tiredness, in patients with rheumatoid arthritis (RA).

**Methods:**

Data from two Phase 3, randomized, placebo-controlled (RA-BUILD; and active-controlled [RA-BEAM]), clinical studies of the efficacy of baricitinib in adults with moderately to severely active RA were used. The psychometric properties of the single-item measure, Severity of Worst Tiredness, were assessed, including test-retest reliability, convergent and discriminant validity, known-groups validity, and responsiveness, using other patient- and clinician-reported outcomes frequently assessed in RA patients.

**Results:**

Test-retest reliability of the Severity of Worst Tiredness was supported through large intraclass correlation coefficients (0.89 ≤ ICC ≤ 0.91). Moderate-to-large correlations were observed between this patient-reported outcome (PRO) and other related patient- and clinician-reported assessments of RA symptoms and patient functioning, supporting construct validity of the measure (│r│ ≥ 0.41). The instrument also displayed known-groups validity through statistically significant differences between mean values of the Severity of Worst Tiredness defined using other indicators of RA severity. Finally, responsiveness was supported by large and statistically significant differences in change scores from Day 1 to Week 12 for patients comparing responders and nonresponders using the American College of Rheumatology 20 (ACR20) criteria.

**Conclusion:**

The Severity of Worst Tiredness PRO demonstrated adequate reliability, validity, and responsiveness in clinical trials of adults with moderately to severely active RA and is fit for purpose in this patient population.

**Electronic supplementary material:**

The online version of this article (10.1186/s12955-017-0807-5) contains supplementary material, which is available to authorized users.

## Background

Development of a standardized approach to assess key elements of disease activity in rheumatoid arthritis (RA) clinical trials has been the goal of Outcome Measures in Rheumatology Clinical Trials (OMERACT), American College of Rheumatology (ACR), and European League Against Rheumatism (EULAR) groups [1–3]. The core sets of measures developed by these groups include assessments and composite indices that incorporate use of patient-reported outcomes (PROs) (e.g., daily functioning, change in disease activity), as well as clinical measures (e.g., erythrocyte sedimentation rate [ESR]) and clinician’s assessments (e.g., clinician assessment of disease activity), to quantify disease activity and change over time [2]. However, patient-centered research has indicated that key outcomes important to patients were not originally captured by the core sets, such as fatigue, sleep, and general wellness [4], morning stiffness [5], and the patient’s experience of social and psychological challenges and ability to cope [6].

Of these, fatigue is noted as one of the most common symptoms experienced by patients with RA [7]. Fatigue is a frequent and debilitating problem for patients with RA [8] and is second only to pain as the most bothersome patient-reported RA symptom [9]. The burden of fatigue in RA patients is well known, with symptom prevalence estimates ranging from 42% to 90% of patients with RA [7, 10, 11]. There is consistent agreement on the clinical relevance of fatigue and the impact fatigue has on activities of daily living and overall health-related quality of life (HRQOL) in RA [12, 13]. Indeed, both the 2007 Patient Perspective Workshop at OMERACT and the 2008 ACR/EULAR Task Force recommended that all RA clinical trials should update the core set of recommended measures of disease activity and report on fatigue [14, 15], although no specific instruments are endorsed.

Although fatigue in RA is a multidimensional concept [16–18], tiredness is a key component of fatigue. Arthritis Research UK [19] defines *fatigue* as “a feeling of extreme physical or mental tiredness,” and the 13-item Functional Assessment of Chronic Illness Therapy-Fatigue (FACIT-F) Scale—a widely used measure of fatigue across many diseases, including RA—has six items that address this key component with “tired” in their wordings (e.g., “I feel tired,” “I have trouble starting things because I am tired,” “I have trouble finishing things because I am tired”). There is wide variation in how patients use the word *fatigue* when describing their symptom experience, with terms like “physical and mental tiredness” commonly associated with fatigue [20].

Moreover, qualitative interviews with RA patients that focused on the development of a new PRO demonstrated that “tiredness” is a more commonly used term to describe this symptom experience than “fatigue” [21]. Specifically, these one-on-one interviews were designed to explore and better understand the terminology RA patients most often use to report this burdensome symptom. In these interviews, the majority of participants (*n* = 20, 71%) used “tiredness” to describe their RA symptom experiences, whereas fewer (*n* = 13, 46%) mentioned “fatigue” [21].

Despite the recommendations for the need to assess this chronic aspect of RA, there is currently no commonly used and well-validated instrument to assess the patient’s experience of this symptom in RA clinical trials [11]. To address this need, a daily electronic PRO diary single-item measure was created to assess the severity of worst tiredness from the patient’s perspective. To develop this single-item measure, referred to as Severity of Worst Tiredness, a targeted literature review and interviews with healthcare providers were conducted in order to ascertain the appropriate terminology to be used for the measure. In addition, qualitative concept elicitation and cognitive debriefing interviews with RA patients were conducted, to ensure that the content of the scale was being accurately captured by the instrument, as well as to confirm that the measure is relevant, easy to use, and easy to understand by patients with RA [21]. This supported the content validity of Severity of Worst Tiredness by confirming the relevance of tiredness as an RA symptom and the appropriateness of the term “tiredness” to describe this symptom. However, although the content validity of Severity of Worst Tiredness was demonstrated, the psychometric properties of the measure have not yet been assessed.

The purpose of the present study is to assess the psychometric properties (i.e., reliability, validity, and responsiveness) of the Severity of Worst Tiredness PRO in patients with moderately to severely active RA who participated in two Phase 3 clinical trials, RA-BEAM and RA-BUILD, for baricitinib.

## Methods

### Patient population

#### RA-BEAM

RA-BEAM (*N* = 1305) was a randomized, double-blind, double-dummy, placebo- and active-controlled, parallel-arm, 52-week study in patients aged ≥18 years with active RA (≥6/68 tender and ≥6/66 swollen joints; serum high-sensitivity C-reactive protein [hsCRP] ≥6 mg/L) with an inadequate response to methotrexate (MTX). The study was designed to assess improvements in disease activity, structural preservation, and PROs, including physical function, safety, and tolerability with oral baricitinib 4 mg once daily. Full details regarding the conduct of the study, as well as the primary efficacy and safety outcomes of this study have been reported previously [22].

#### RA-BUILD

RA-BUILD (*N* = 684) was a randomized, double-blind, placebo-controlled, parallel-group 24-week study in patients aged ≥18 years with active RA (≥6/68 tender and ≥6/66 swollen joints; hsCRP ≥3.6 mg/L [upper limit of normal 3.0 mg/L]) and an insufficient response (despite prior therapy) or intolerance to ≥1 csDMARDs. The study was designed to assess improvements in disease activity, structural preservation, and PROs, including physical function, safety, and tolerability with oral baricitinib 2 and 4 mg once daily. Full details regarding the conduct of the study, as well as the primary efficacy and safety outcomes of this study have been reported previously [23].

For both studies, the current analysis is on data between Weeks 0 to 12, utilizing other PRO and clinician-reported indicators of RA symptoms and severity assessed in the primary efficacy studies of RA-BEAM and RA-BUILD. Both studies were conducted with informed consent, under institutional review board approval, and in accordance with the Declaration of Helsinki (ClinicalTrials.gov number NCT01710358 [RA-BUILD] and NCT01721057 [RA-BEAM]).

### Instruments used in the psychometric analyses

#### Patient-reported outcomes (PROs)

##### Severity of Worst Tiredness, Severity of Morning Joint Stiffness, Severity of Worst Joint Pain, and Duration of Morning Joint Stiffness

Severity of Worst Tiredness, Severity of Morning Joint Stiffness (MJS), and Severity of Worst Joint Pain are all single-item PROs designed to capture the severity of worst tiredness, MJS, and worst joint pain experienced that day, respectively. All three of these PROs are anchored at 0 and 10, where 0 represents “no tiredness,” “no joint stiffness,” or “no joint pain,” and 10 represents “tiredness as bad as you can imagine,” “joint stiffness as bad as you can imagine,” or “joint pain as bad as you can imagine,” respectively. The Duration of MJS is a single-item PRO designed to capture information on self-reported length of time, in minutes, that a patient’s MJS lasted each day. Durations recorded as >12 h (720 min) were censored at 720 min.

For RA-BEAM and RA-BUILD, all four PROs were assessed using a daily electronic diary through Week 12. The Day 1 assessment was the first assessment at the end of the patient’s day after the randomization visit (Week 0, Visit 2). The Week 1 assessment refers to the weekly average values from Days 2 to 8. Assessments at Weeks 2, 4, 8, and 12 refer to weekly average values of the 7 days prior to Weeks 2, 4, 8, and 12 visits, respectively. Recognizing that late shift workers (individuals who work outside of the hours of 9 am until 5 pm) could not complete the electronic diary (at home) at the end of Day 1, the Day 2 assessment (if available) was used to impute missing Day 1 values so that more patients could be included in the psychometric analyses utilizing the Day 1 value.

##### Medical Outcomes Study 36-Item Short Form Health Survey Version 2 Acute (SF-36)

The SF-36 is a generic, 36-item PRO that measures general health status. The SF-36 includes eight domains of health status evaluated over the prior week: physical function, role limitations–physical, bodily pain, general health perceptions, vitality, social function, role limitations–emotional, and mental health. Two component scores, the Physical Component Score (PCS) and the Mental Component Score (MCS), are derived based on the eight domain scores [24]. Domain and component scores are derived using established formulas [24], with higher scores indicating better health status or functioning.

##### Functional Assessment of Chronic Illness Therapy-Fatigue (FACIT-F)

The FACIT-F scale [25] is a brief, 13-item, symptom-specific questionnaire that specifically assesses the self-reported severity of fatigue caused by chronic disease and its impact on daily activities and functioning. A 5-point Likert-type scale (0 = not at all; 1 = a little bit; 2 = somewhat; 3 = quite a bit; 4 = very much) is used. The range of possible scores is 0 to 52, with 0 being the worst possible score (indicating greater fatigue) and 52 the best (indicating lesser fatigue).

##### Health Assessment Questionnaire-Disability Index (HAQ-DI)

The HAQ-DI assesses patient physical function or disability. The HAQ-DI contains 24 questions that query the degree of difficulty a person has in accomplishing tasks in eight functional areas (dressing, arising, eating, walking, hygiene, reaching, gripping, and activities). Responses in each functional area are scored from 0, indicating “no difficulty” in that area, to 3, indicating “inability to perform a task” in that area. The HAQ-DI total score, ranging from 0 to 3 (higher values indicate worse functioning), is obtained by summing the highest score within each functional area and dividing by the number of functional areas answered [26].

##### Quick Inventory of Depressive Symptomatology Self-Rated-16 (QIDS-SR_16_)

The QIDS-SR_16_ is a 16-item PRO intended to assess the existence and severity of symptoms of depression as listed in the American Psychiatric Association’s Diagnostic and Statistical Manual of Mental Disorders, 4th Edition [27]. Patients were asked to consider each statement as it relates to the way they have felt for the past 7 days. There is a unique 4-point ordinal scale for each item, with scores ranging from 0 to 3 reflecting increasing depressive symptoms as the item score increases. The instrument measures nine core symptom domains that are used to define a depressive episode: sad mood; concentration; self-criticism; suicidal ideation; interest; energy/fatigue; sleep disturbance; decrease or increase in appetite or weight; and psychomotor agitation or retardation. The QIDS-SR_16_ total score is derived as the sum of the scores across the nine scale domains.

##### Patient’s assessment of pain

Patient’s pain was assessed at each study visit with the use of a 0–100 mm visual analogue scale (VAS), with higher scores indicating more severe pain. Specifically, patients were asked, “How much pain are you currently having because of your rheumatoid arthritis?”

##### Patient’s Global Assessment of Disease Activity (PtGA)

The PtGA was assessed at each study visit and is recorded on a 0–100 mm VAS, with higher scores indicating more active RA.

#### Clinician-reported assessments

##### Physician’s Global Assessment of Disease Activity (PhGA)

The PhGA was assessed at each study visit and is recorded on a 0–100 mm VAS, with higher scores indicating more active RA.

#### Clinical sign and symptom measures

##### American College of Rheumatology 20 (ACR20)

An ACR20 response (i.e., a binary variable indicating achieving or not achieving a response) was measured at each study visit and is defined as at least a 20% improvement from baseline in both tender joint count (TJC) (0 to 68) and swollen joint count (SJC) (0 to 66), and in at least three of the following five assessments: patient’s assessment of pain, PtGA, PhGA, HAQ-DI, and hsCRP.

#### Clinical Disease Activity Index (CDAI)

The CDAI is a tool for measurement of disease activity in RA that integrates measures of physical examination, patient self-assessment, and evaluator assessment [28]. The CDAI was assessed at each study visit and is calculated by adding together scores from the following assessments: number of swollen joints (0 to 28), number of tender joints (0 to 28), PtGA on a VAS (0 to 10 cm), and PhGA on a VAS (0 to 10 cm). Total scores are calculated using established formulas [28]. Thresholds have been established for the CDAI (remission: ≤2.8; low disease activity: >2.8 to ≤10; moderate disease activity: >10 to ≤22; high disease activity: >22 to ≤76) [29].

#### Disease activity score (28 joints) (DAS28)

The DAS28 is a composite score that is based on a 28-joint count (both TJC 0 to 28 and SJC 0 to 28), hsCRP or ESR, and PtGA and was measured at each study visit. Total scores are calculated using established formulas [30]. Patients can be categorized into four groups (remission: <2.6; low disease activity: ≥2.6 to ≤3.2; moderate disease activity: >3.2 to ≤5.1; high disease activity: >5.1).

### Statistical analyses

#### Reliability (test-retest)

For the assessment of test-retest reliability (which is used to assess if instrument scores are reproducible across time), stable patients were defined as patients with ≤5 point difference [31] on the 0 to 100 PtGA between each assessment period, including between Weeks 1 and 2 and again between Weeks 4 and 8. Intraclass correlation coefficients (ICCs) were calculated between Weeks 1 and 2 and again between Weeks 4 and 8 to evaluate test-retest reliability. An ICC of ≥0.70 was considered good agreement [32].

### Convergent and discriminant validity (construct validity)

Construct validity is the degree to which scores from one measure are related to those of other measures in a manner that is theoretically consistent. Pearson correlations at Day 1 and Week 12 were used to assess for the construct validity of Severity of Worst Tiredness. Correlations were calculated at Day 1 and Week 12 between Severity of Worst Tiredness and the scores of other clinical/PRO endpoints: Severity of MJS, Severity of Worst Joint Pain, Duration of MJS, SF-36 domain and component scores, FACIT-F, HAQ-DI, QIDS-SR_16_, patient’s assessment of pain, PtGA, TJC28, SJC28, PhGA, and hsCRP. The strength of the correlations were interpreted using Cohen’s conventions, where a correlation >0.5 is large, 0.3 to 0.5 is moderate, 0.1 to <0.3 is small, and <0.1 is insubstantial [33].

It was hypothesized that moderate or large correlations supporting convergent validity at Day 1 and Week 12 would be demonstrated between Severity of Worst Tiredness, and PRO instruments measuring concepts related to tiredness or fatigue (FACIT-F, SF-36 Vitality), other RA pain-like symptoms (Severity of MJS, SF-36 Bodily Pain, Severity of Worst Joint Pain, patient’s assessment of pain), their impact on functioning (SF-36 Social Functioning, SF-36 Physical Functioning, HAQ-DI), and clinician-reported/laboratory assessments of disease activity (TJC28, SJC28, PhGA, and hsCRP). Discriminant validity was assessed by Pearson correlations at Day 1 and at Week 12 between Severity of Worst Tiredness, and PROs measuring distally related concepts (SF-36 MCS, SF-36 Role Emotional, QIDS-SR_16_) where small correlations were hypothesized.

### Known-groups validity

Known-groups validity tests seek to demonstrate differences between two or more groups known to differ on the underlying construct [34]. An analysis of variance (ANOVA) model was used for the assessment of known-groups validity at Day 1 and Week 4 to distinguish mean Severity of Worst Tiredness between subgroups defined by the DAS28-ESR thresholds (<2.6; ≥2.6 and ≤3.2; >3.2 and ≤5.1; and >5.1) and CDAI (0.0 to ≤2.8; >2.8 to ≤10; >10 to ≤22; and >22 to ≤76). The Scheffé adjustment was used for multiple comparisons. When subgroup sample sizes were small (i.e., <5% of the total sample size for the subgroup), subgroups were combined.

### Responsiveness

Responsiveness, or the ability of the instrument to detect change over time [35], of Severity of Worst Tiredness was evaluated using an analysis of covariance (ANCOVA) methodology to assess significant differences in mean change in Severity of Worst Tiredness from Day 1 to Week 12 between ACR20 responders and nonresponders at Week 12, controlling for Day 1 Severity of Worst Tiredness. A parallel analysis was also conducted to assess responsiveness using disease activity as measured by DAS28-hsCRP at Week 12, using the following subgroups: DAS28-hsCRP <2.6, DAS28-hsCRP ≥2.6 and DAS28-hsCRP ≤3.2, and DAS28-hsCRP >3.2. An overall statistically significant difference (*p* < 0.05) with statistically significant subgroup comparisons was hypothesized.

### Handling of missing data

Only patients with Day 1 data were included in analyses at Day 1. For analyses of data at Week 12, scores collected in the 7 days prior to the Week 12 visit date were used. If there were fewer than 4 nonmissing assessments, the 7-day window was shifted back in time (toward baseline) one day at a time until there were 4 nonmissing assessments available in the 7-day window. Then, the average of the 4 assessments was used in the Week 12 analysis.

## Results

Baseline demographics for the total modified intent-to-treat population, patients with Day 1 diary scores, and patients with Week 12 diary scores are provided in Table 1. Scores for all other patient- and clinician-completed assessments, as well as clinical sign and symptom measures, are found in Table 2.

**Table 1:** Patient Demographic and Disease Characteristics Patients with Electronic Diary Assessments at Day 1 and Patients with Week 12 Electronic Diary Assessments (mITT Population) for RA-BEAM and RA-BUILD

Characteristics	RA-BEAM			RA-BUILD		
	Total mITT Population (*n* = 1305)	Patients with Day 1 Diary Scores (*n* = 537)	Patients with Week 12 Diary Scores^a^ (*n* = 1281)	Total mITT Population (*n* = 684)	Patients with Day 1 Diary Scores (*n* = 312)	Patients with Week 12 Diary Scores^a^ (*n* = 666)
Age (years)						
Mean (SD)	53.3 (12.1)	53.2 (12.3)	53.2 (12.0)	51.8 (12.3)	51.5 (12.3)	51.8 (12.3)
Gender, n (%)						
Female	1009 (77.3%)	414 (77.1%)	989 (77.2%)	560 (81.9%)	262 (84.0%)	546 (82.0%)
Race, n (%)						
White	818 (62.7%)	422 (78.6%)	801 (62.5%)	457 (66.8%)	254 (81.4%)	444 (66.7%)
Black or African American	10 (0.8%)	5 (0.9%)	9 (0.7%)	28 (4.1%)	16 (5.1%)	27 (4.1%)
Asian	392 (30.0%)	45 (8.4%)	388 (30.3%)	180 (26.3%)	31 (9.9%)	176 (26.4%)
American Indian or Alaska Native	63 (4.8%)	49 (9.1%)	61 (4.8%)	14 (2.0%)	8 (2.6%)	14 (2.1%)
Native Hawaiian or Other Pacific Islander	0 (0.0%)	0 (0.0%)	0 (0.0%)	1 (0.1%)	0 (0.0%)	1 (0.1%)
Multiple	21 (1.6%)	16 (3.0%)	21 (1.6%)	3 (0.4%)	2 (0.6%)	3 (0.5%)
Missing	1 (0.1%)	0 (0.0%)	1 (0.1%)	1 (0.1%)	1 (0.3%)	1 (0.2%)
Years from RA Diagnosis						
Mean (SD) [Min, Max]	8.7 (8.2) [0–56]	8.7 (8.0) [0–40]	8.6 (8.1) [0–56]	6.3 (7.3) [0–53]	6.2 (6.5) [0–41]	6.4 (7.4) [0–53]

Abbreviations: Max = maximum; Min = minimum; mITT = modified intent-to-treat; n = number of patients in the specified category; RA = rheumatoid arthritis; SD = standard deviation

^a^Daily average of seven days preceding visit that contained at least 4 measurements

**Table 2:** Instrument Scores at Day 1 and Week 12 for RA-BEAM and RA-BUILD

Instrument	RA-BEAM^a^		RA-BUILD^b^	
	Day 1	Week 12	Day 1	Week 12
Severity of Worst Tiredness, Mean (SD)	5.8 (2.1)	4.0 (2.3)	6.0 (2.1)	4.1 (2.4)
Duration of MJS, Mean (SD)	152.8 (180.8)	96.7 (144.7)	160.7 (174.8)	103.5 (147.5)
Severity of MJS, Mean (SD)	5.8 (2.2)	3.5 (2.4)	5.7 (2.1)	3.7 (2.4)
Severity of Worst Joint Pain, Mean (SD)	6.1 (2.0)	4.0 (2.3)	6.0 (2.1)	4.2 (2.4)
Patient’s Assessment of Pain, Mean (SD)	60.8 (22.3)	34.9 (24.1)	58.0 (22.1)	36.5 (24.9)
PtGA, Mean (SD)	62.4 (21.8)	36.3 (23.5)	60.7 (21.1)	37.7 (24.0)
SF-36				
Role Physical Domain, Mean (SD)	35.5 (10.4)	41.8 (10.2)	35.3 (9.2)	40.7 (9.6)
Bodily Pain Domain, Mean (SD)	34.7 (7.8)	42.5 (8.7)	34.9 (7.6)	41.9 (9.0)
General Health Domain, Mean (SD)	36.8 (8.5)	41.6 (8.9)	36.8 (8.3)	42.0 (9.4)
Social Functioning Domain, Mean (SD)	40.8 (11.6)	45.4 (10.5)	40.2 (11.2)	44.7 (10.3)
Vitality Domain, Mean (SD)	43.7 (10.0)	49.4 (9.8)	41.9 (10.1)	48.4 (10.0)
Role Emotional Domain, Mean (SD)	41.1 (12.6)	45.5 (10.9)	40.9 (12.3)	44.8 (11.2)
Mental Health Domain, Mean (SD)	43.0 (11.3)	47.1 (10.7)	42.9 (11.6)	47.1 (11.3)
Physical Functioning Domain, Mean (SD)	32.2 (10.4)	38.7 (10.9)	32.2 (10.2)	38.2 (10.9)
Mental Component Score, Mean (SD)	46.4 (11.8)	49.8 (10.8)	45.7 (11.8)	49.3 (11.1)
Physical Component Score, Mean (SD)	32.3 (8.6)	39.4 (9.3)	32.3 (8.3)	38.8 (9.4)
FACIT-F Total Score, Mean (SD)	28.2 (10.9)	36.8 (9.6)	26.8 (11.2)	35.5 (10.1)
QIDS-SR_16_ Total Score, Mean (SD)	8.0 (5.0)	5.9 (4.2)	8.4 (4.9)	6.2 (4.3)
HAQ-DI Total Score, Mean (SD)	1.56 (0.68)	1.03 (0.70)	1.52 (0.61)	1.03 (0.66)
PhGA, Mean (SD)	65.0 (16.9)	31.0 (21.7)	63.5 (17.4)	33.9 (22.4)
CDAI, Mean (SD)	37.83 (12.55)	18.68 (13.54)	36.05 (12.12)	18.04 (13.15)
DAS28-ESR, Mean (SD)	6.43 (0.97)	4.62 (1.46)	6.22 (0.97)	4.50 (1.37)
DAS28-hsCRP, Mean (SD)	5.73 (0.94)	3.93 (1.39)	5.55 (0.91)	3.84 (1.35)

Abbreviations: CDAI = Clinical Disease Activity Index; DAS28 = Disease Activity Score modified to include the 28 diarthrodial joint count; ESR = erythrocyte sedimentation rate; FACIT-F = Functional Assessment of Chronic Illness Therapy-Fatigue; HAQ-DI = Health Assessment Questionnaire-Disability Index; hsCRP = high-sensitivity C-reactive protein; *n* = number of patients; MJS = morning joint stiffness; PhGA = Physician’s Global Assessment of Disease Activity; PtGA = Patient’s Global Assessment of Disease Activity; QIDS-SR_16_ = Quick Inventory of Depressive Symptomatology Self-Rated-16; SD = standard deviation; SF-36 = 36-Item Short Form Health Survey version 2 with Acute recall

^a^Daily diary responses were *n* = 537(Day 1) and *n* = 1281 (Week 12); nondiary assessments had approximately *n* = 1301 (Day 1) and *n* = 1250 (Week 12)

^b^Daily diary responses were *n* = 310 (Day 1) and *n* = 666 (Week 12); nondiary assessments had approximately *n* = 680 (Day 1) and *n* = 644 (Week 12)

A large amount of missing data was present at the Day 1 assessment period (Tables 1 and 2). These missing data were due to multiple reasons as shown in Additional file 1: Table S1, such as the diary device alarm not sounding until the following day or the diary device being given to the patient after Day 1. Sensitivity analyses with the imputation for missing data at Day 1 (*n* = 1041 for RA-BEAM and *n* = 563 for RA-BUILD, respectively) were conducted and demonstrated similar findings to the results presented here.

### Reliability (test-retest)

From Weeks 1 to 2, ICCs for weekly mean severity of worst tiredness ranged from 0.90 to 0.91 (RA-BEAM *n* = 412; RA-BUILD *n* = 185) and from 0.89 to 0.91 from Week 4 to Week 8 (RA-BEAM *n* = 417; RA-BUILD *n* = 215). These values provide evidence for substantial test-retest reliability among stable patients.

### Convergent and discriminant validity

Results supporting convergent validity of Severity of Worst Tiredness in terms of its relationship with other clinical outcome assessments are presented in Table 3 at Day 1 and Table 4 at Week 12. At Day 1 in RA-BEAM and RA-BUILD, moderate-to-large associations between Severity of Worst Tiredness and other assessments measuring similar tiredness-like patient states were demonstrated. These associations were found to be large at Week 12 in RA-BEAM and RA-BUILD, including the FACIT-F (*r* = −0.60 in both studies) and SF-36 Vitality (*r* = −0.52 and −0.51). In addition, Severity of Worst Tiredness also demonstrated moderate-to-large associations with measures of other RA symptoms of pain and stiffness at Day 1. These associations increased at Week 12 in RA-BEAM and RA-BUILD, respectively, including SF-36 Bodily Pain (*r* = −0.51 and −0.52), Worst Joint Pain (*r* = 0.82 and 0.83), Severity of MJS (*r* = 0.79 and 0.77), and patient’s assessment of pain (*r* = 0.69 and 0.65). The Severity of Worst Tiredness also demonstrated moderate-to-large associations with concepts related to patient physical and social functioning at Day 1 that increased at Week 12, in RA-BEAM and RA-BUILD, respectively, including SF-36 Social Functioning (*r* = −0.43 and −0.44), SF-36 Physical Functioning (*r* = −0.43 and −0.41), and HAQ-DI (*r* = 0.49 and 0.46). These findings provide support for the convergent validity of Severity of Worst Tiredness in patients with moderately to severely active RA.

**Table 3:** Pearson Correlations between Severity of Worst Tiredness and Other Instruments in RA-BEAM and RA-BUILD at Day 1

Instruments	Severity of Worst Tiredness			
	RA-BEAM		RA-BUILD	
	n	*r*	n	*r*
Day 1				
Duration of MJS	537	0.21***	311	0.09
Severity of MJS	537	0.66***	311	0.52***
Severity of Worst Joint Pain	537	0.70***	310	0.59***
SF-36				
Role Physical Domain	535	−0.44***	311	−0.39***
Bodily Pain Domain	535	−0.56***	311	−0.46***
General Health Domain	535	−0.37***	311	−0.33***
Social Functioning Domain	535	−0.44***	311	−0.35***
Vitality Domain	535	−0.49***	311	−0.53***
Role Emotional Domain	535	−0.35***	311	−0.21***
Mental Health Domain	535	−0.39***	311	−0.31***
Physical Functioning Domain	535	−0.47***	311	−0.38***
Mental Component Score	535	−0.38***	311	−0.31***
Physical Component Score	535	−0.47***	311	−0.41***
FACIT-F Total Score	535	−0.57***	311	−0.60***
HAQ-DI Total Score	535	0.48***	311	0.37***
QIDS-SR_16_ Total Score	535	0.40***	311	0.37***
Patient’s Assessment of Pain	535	0.43***	311	0.29***
PtGA	535	0.45***	311	0.30***
Tender Joint Count 28	537	0.21***	311	0.30***
Swollen Joint Count 28	537	0.18***	311	0.20***
PhGA	535	0.21***	304	0.20***
hsCRP	535	0.37***	311	0.33***

Abbreviations: FACIT-F = Functional Assessment of Chronic Illness Therapy-Fatigue; HAQ-DI = Health Assessment Questionnaire-Disability Index; hsCRP = high-sensitivity C-reactive protein; MJS = morning joint stiffness; *n* = number of patients; PtGA = Patient’s Global Assessment of Disease Activity; PhGA = Physician’s Global Assessment of Disease Activity; QIDS-SR_16_ = Quick Inventory of Depressive Symptomatology Self-Rated-16; SF-36 = 36-Item Short Form Health Survey version 2 with Acute recall

Pearson correlation coefficients with significance using *p*-values denoted as: * ≤ 0.05, ** ≤ 0.01, *** ≤ 0.001

**Table 4:** Pearson Correlations between Severity of Worst Tiredness and Other Instruments in RA-BEAM and RA-BUILD at Week 12

Instruments	Severity of Worst Tiredness			
	RA-BEAM		RA-BUILD	
	n	*r*	n	*r*
Week 12^a^				
Duration of MJS	1281	0.32***	666	0.38***
Severity of MJS	1281	0.79***	666	0.77***
Severity of Worst Joint Pain	1281	0.82***	666	0.82***
SF-36				
Role Physical Domain	1233	−0.45***	632	−0.42***
Bodily Pain Domain	1233	−0.51***	632	−0.52***
General Health Domain	1233	−0.39***	632	−0.38***
Social Functioning Domain	1233	−0.43***	632	−0.44***
Vitality Domain	1233	−0.52***	632	−0.51***
Role Emotional Domain	1233	−0.41***	632	−0.32***
Mental Health Domain	1233	−0.40***	632	−0.34***
Physical Functioning Domain	1233	−0.43***	632	−0.41***
Mental Component Score	1233	−0.40***	632	−0.34***
Physical Component Score	1233	−0.45***	632	−0.45***
FACIT-F Total Score	1233	−0.60***	632	−0.60***
HAQ-DI Total Score	1233	0.49***	632	0.46***
QIDS-SR_16_ Total Score	1231	0.37***	632	0.37***
Patient’s Assessment of Pain	1233	0.69***	632	0.65***
PtGA	1233	0.64***	632	0.62***
Tender Joint Count 28	1231	0.32***	630	0.40***
Swollen Joint Count 28	1231	0.21***	630	0.23***
PhGA	1224	0.35***	627	0.38***
hsCRP	1220	0.46***	623	0.49***

Abbreviations: FACIT-F = Functional Assessment of Chronic Illness Therapy-Fatigue; HAQ-DI = Health Assessment Questionnaire-Disability Index; hsCRP = high-sensitivity C-reactive protein; MJS = morning joint stiffness; *n* = number of patients; PtGA = Patient’s Global Assessment of Disease Activity; PhGA = Physician’s Global Assessment of Disease Activity; QIDS-SR_16_ = Quick Inventory of Depressive Symptomatology Self-Rated-16; SF-36 = 36-Item Short Form Health Survey version 2 with Acute recall

Pearson correlation coefficients with significance using *p*-values denoted as: * ≤ 0.05, ** ≤ 0.01, *** ≤ 0.001

^a^Daily average of seven days preceding visit that contained at least 4 measurements

Small-to-moderate correlations were observed between Severity of Worst Tiredness and SF-36 MCS (*r* = −0.38 and −0.31) and SF-36 Role Emotional (*r* = −0.35 and −0.21) at Day 1, as well as QIDS-SR_16_ (*r* = 0.40 to 0.37) in RA-BEAM and RA-BUILD, respectively, indicating that these assessments measure more distally related constructs as hypothesized.

### Known-groups validity

Because small sample sizes in the lower DAS28-ESR subgroups at Day 1 (i.e., <5% of the sample in each score category), patients were categorized into two subgroups: ≤5.1 and >5.1 (Table 5). At Day 1 in RA-BEAM and RA-BUILD, patients with higher DAS28-ESR scores reported a significantly greater Severity of Worst Tiredness score than those patients with lower DAS28-ESR scores (Table 5). Similar results were found for both studies at Week 4.

**Table 5:** Known-Groups Validity of Severity of Worst Tiredness Using DAS28-ESR Subgroups at Day 1 and Week 4

PRO/Study	DAS28-ESR Category		*p*-value
	≤5.1 N, Mean (SD)	>5.1 N, Mean (SD)	
Severity of Worst Tiredness Day 1			
RA-BEAM	39, 4.7 (2.6)	496, 5.9 (2.1)	0.001^a^
RA-BUILD	36, 5.0, (1.9)	273, 6.2 (2.0)	0.002^a^
Severity of Worst Tiredness Week 4			
RA-BEAM	619, 3.6 (2.1)	587, 5.3 (1.9)	0.001^a^
RA-BUILD	327, 3.7 (2.0)	286, 5.5 (2.0)	0.001^a^

Abbreviations: DAS28 = Disease Activity Score modified to include the 28 diarthrodial joint count; ESR = erythrocyte sedimentation rate; SD = standard deviation;

^a^ *p*-value based on a comparison of mean values using ANOVA

Similarly, because of small sample sizes, patients were categorized into two subgroups based on the CDAI at Day 1 (0.0 to ≤22.0 and >22.0 to ≤76.0) and three subgroups at Week 4 (0.0 to ≤10.0, >10.0 to ≤22.0, and >22.0 to ≤76.0). At Day 1, patients in the higher CDAI score subgroup experienced a significantly greater Severity of Worst Tiredness in both RA-BEAM and RA-BUILD than those patients in the lower CDAI score subgroup (Table 6). Similar results were found for both studies at Week 4 (Table 7). These findings provide evidence that Severity of Worst Tiredness is able to distinguish between known groups based on disease severity.

**Table 6:** Known-Groups Validity of Severity of Worst Tiredness Using CDAI Subgroups at Day 1

PRO/Study	CDAI Category		*p*-value
	0.0 to ≤ 22.0 N, Mean (SD)	>22.0 to ≤ 76.0 N, Mean (SD)	
Severity of Worst Tiredness Day 1			
RA-BEAM	36, 4.9 (2.6)	497, 5.9 (2.1)	0.007^a^
RA-BUILD	26, 4.8 (2.1)	278, 6.1 (2.0)	0.016^a^

Abbreviations: SD = standard deviation; CDAI = Clinical Disease Activity Index

^a^ *p*-value based on a comparison of mean values using ANOVA

**Table 7:** Known-Groups Validity of Severity of Worst Tiredness Using CDAI Subgroups at Week 4

PRO/Study	CDAI Category			*p*-value^a^	Comparisons^b^
	0.0 to ≤ 10.0 N, Mean (SD)	>10.0 to ≤ 22.0 N, Mean (SD)	>22.0 to ≤ 76.0 N, Mean (SD)		
Severity of Worst Tiredness Week 4					
RA-BEAM	220, 2.9 (2.0)	426, 4.2 (2.1)	567, 5.1 (2.0)	0.001	a: 0.001, b: 0.001, c: 0.001
RA-BUILD	134, 3.1 (2.0)	209, 4.1 (1.9)	275, 5.6 (2.0)	0.001	a: 0.001, b: 0.001, c: 0.001

Abbreviations: SD = standard deviation; CDAI = Clinical Disease Activity Index

^a^p-value based on a comparison of mean values using ANOVA

^b^Note for multiple comparison using Scheffé adjustment: a: 0.0 to ≤10.0 versus >10.0 to ≤22.0; b: 0.0 to ≤10.0 versus >22.0 to ≤76.0; c: >10.0 to ≤22.0 versus >22.0 to ≤76.0

### Responsiveness

The responsiveness of Severity of Worst Tiredness was supported through large and statistically significant differences in mean change from Day 1 to Week 12 in Severity of Worst Tiredness between ACR20 responders and non-responders (Table 8). Similar findings supporting responsiveness of Severity of Worst Tiredness were seen when using DAS28-hsCRP as an anchor. Pairwise comparisons assessing for significant differences in mean change between DAS28-hsCRP subgroups of <2.6 versus ≥3.2 (*p* = 0.001 for both studies), and ≥2.6 and <3.2 versus ≥3.2 (*p* = 0.001 for both studies) were statistically significant (Table 8). However, the comparisons between change scores for subgroups <2.6 versus ≥2.6 and <3.2 were not statistically significant for either study.

**Table 8:** Change in Severity of Worst Tiredness from Day 1 to Week 12 among ACR20 and DAS28-hsCRP Groups

PRO/Study/Statistics	ACR20 Category at Week 12		*p*-value	DAS28-hsCRP Groups at Week 12^b^			Comparisons^c^
	Responder^a^	Nonresponder^a^		DAS28-hsCRP < 2.6	2.6 ≤ DAS28-hsCRP < 3.2	DAS28-hsCRP ≥ 3.2	
RA-BEAM; N, Mean (SD)	326, −2.5 (2.5)	211, −1.0 (2.1)	0.001	88, −3.1 (2.7)	79, −2.4 (2.4)	368, −1.5 (2.3)	a: 0.069; b: 0.001; c: 0.001
RA-BUILD; N, Mean (SD)	174, −2.6 (2.6)	137, −0.9 (2.0)	0.001	61, −2.9 (2.8)	33, −2.6 (3.2)	214, −1.5 (2.2)	a: 0.846; b: 0.001; c: 0.001

Abbreviations: ACR20 = 20% improvement in American College of Rheumatology criteria; DAS28 = Disease Activity Score modified to include the 28 diarthrodial joint count; hsCRP = high-sensitivity C-reactive protein; SD = standard deviation

^a^Responder: Achievement of ACR20 criteria at Week 12; Nonresponder: Failure to achieve ACR20 criteria at Week 12

^b^Missing DAS28-hsCRP at Week 12 was imputed using modified baseline observation carried forward

^c^Note for multiple comparison: a: <2.6 vs. ≥2.6 and <3.2; b: <2.6 vs. ≥3.2; c: ≥2.6 and <3.2 vs. ≥3.2

^d^Daily average of seven days preceding visit that contained at least 4 measurements

## Discussion

An investigation into the psychometric properties of Severity of Worst Tiredness PRO using data from patients with moderately to severely active RA provided support for the reliability, validity, and responsiveness of this measure. Analyses of test-retest reliability indicated strong agreement in Severity of Worst Tiredness scores across two assessment periods in stable patients. The construct (convergent and divergent) validity of Severity of Worst Tiredness was also supported, as a priori hypotheses of the associations between Severity of Worst Tiredness and related PROs, clinician-reported measures, and laboratory assessments were supported at Day 1 and Week 12. Using the DAS28-ESR and CDAI as indicators of known clinical status, known-groups validity was supported as mean Severity of Worst Tiredness values were significantly different between predefined groups. Lastly, Severity of Worst Tiredness demonstrated responsiveness to change from Day 1 to Week 12 when defining responders using the ACR20 or DAS28-hsCRP as an anchor.

Patients have identified tiredness/fatigue as a bothersome and debilitating disease-related symptom [8, 9], and despite improved treatment options for other RA symptoms, improvement in fatigue continues to be noted as an unmet need for patients with RA [18]. This was recently demonstrated in an analysis of data from the Leiden Early Arthritis Clinic cohort of patients with RA [36]. Cohort inclusion occurred when RA was confirmed at physical examination and symptom duration was <2 years (early RA). Early RA treatment strategies evolved over time, such that initial treatment for patients enrolled from 1993 to 1995 was nonsteroidal anti-inflammatory drugs (NSAIDs) (DMARDs were used later in treatment); patients enrolled from 1996 to 1998 were treated with non-MTX DMARDs (usually hydroxychloroquine or sulfasalazine); or patients enrolled from 1999 to 2007 were treated with MTX. A longitudinal study of 626 patients from these three cohorts demonstrated that despite improved treatment strategies over time associated with less severe radiographic progression in RA, there was no effect on fatigue severity over many years of treatment in early RA patients (*p* = 0.96) [36]. The authors concluded that a reliable and valid PRO measure of this symptom is an important tool to aid clinicians in treating patients with RA, thereby facilitating doctor-patient communication to improve the quality of patient care, contribute to better patient outcomes, and help to address this need [37]. Thus, the Severity of Worst Tiredness PRO addresses this unmet need. Given the increasing use of electronic PRO diaries in clinical settings, this instrument could be utilized in a clinical practice where patients are asked to report their worst tiredness symptom daily, thus enhancing the dialogue between patients and care providers. The reliability and ability to detect change over time has been demonstrated and further supports the use of this instrument as a simple, single-item instrument of RA-related tiredness.

Although Severity of Worst Tiredness did display strong evidence of reliability, validity, and responsiveness, a key limitation to this study is the missing data at the Day 1 assessment. These missing data were due to multiple reasons such as the missed alarms. However, sensitivity analyses after imputing missing Day 1 Severity of Worst Tiredness scores were conducted and all study conclusions remained the same. The timespan of the baseline assessment is also a limitation in that it only consisted of one study day’s data versus the average of up to the 7 days of assessments, as used in the Week 12 endpoint.

## Conclusion

The results from the present study demonstrate that the single-item, daily measure, Severity of Worst Tiredness, is suitable to validly and reliably measure a key symptom of RA that is important to patients with moderately to severely active RA.

## Additional file

Additional file 1: Table S1. Reasons for Missing Diary Data at Day 1 for RA-BEAM and RA-BUILD. (DOC 29 kb)

## Abbreviations

ACR: American College of Rheumatology
ACR20: 20% improvement in American College of Rheumatology criteria
ANCOVA: analysis of covariance
ANOVA: analysis of variance
csDMARD: conventional synthetic DMARD
DAS28: Disease Activity Score modified to include the 28 diarthrodial joint count
DMARD: disease-modifying antirheumatic drug
ESR: erythrocyte sedimentation rate
EULAR: European League Against Rheumatism
FACIT-F: Functional Assessment of Chronic Illness Therapy-Fatigue
HAQ-DI: Health Assessment Questionnaire-Disability Index
HRQOL: health-related quality of life
hsCRP: high-sensitivity C-reactive protein
ICC: intraclass correlation coefficient
MCS: Mental Component Score
MJS: Morning Joint Stiffness
NSAID: nonsteroidal anti-inflammatory drug
OMERACT: Outcome Measures in Rheumatology Clinical Trials
PCS: Physical Component Score
PhGA: Physician’s Global Assessment of Disease Activity
PRO: patient-reported outcome
PtGA: Patient’s Global Assessment of Disease Activity
QIDS-SR_16_: Quick Inventory of Depressive Symptomatology Self-Rated-16
RA: rheumatoid arthritis
SJC: swollen joint count
TJC: tender joint count
TNF: tumor necrosis factor
VAS: visual analogue scale
