# Supplementary material for: Psychometric properties of the single-item measure, severity of worst tiredness, in patients with moderately to severely active rheumatoid arthritis
Source: Health Qual Life Outcomes. 2017 Dec 6;15:237. doi: 10.1186/s12955-017-0807-5 (PMC5718135; doi:10.1186/s12955-017-0807-5)
Supplement: Additional file 1: Table S1. — Reasons for Missing Diary Data at Day 1 for RA-BEAM and RA-BUILD. (DOC 29 kb) [file 12955_2017_807_MOESM1_ESM.doc]

Supplementary Table S1. Reasons for Missing Diary Data at Day 1 for RA-BEAM and RA-BUILD

| Reason, n (%) | RA-BEAM N=768 | RA-BUILD N=372 |
| --- | --- | --- |
| Device never given to patient | 17 (2.2) | 12 (3.2) |
| Device given to patient after baseline (Day 1) | 130 (16.9) | 57 (15.3) |
| Missed alarms | 97 (12.6) | 48 (12.9) |
| Low battery | 82 (10.7) | 25 (6.7) |
| First alarm next day | 256 (33.3) | 208 (55.9) |
| User abandoned or delayed report past window | 171 (22.3) | 21 (5.6) |
| Other | 15 (2) | 1 (0.3) |
